# Supplementary material for: A new leptoceratopsid dinosaur from Maastrichtian-aged deposits of the Sustut Basin, northern British Columbia, Canada
Source: PeerJ. 2019 Nov 7;7:e7926. doi: 10.7717/peerj.7926 (PMC6842559; doi:10.7717/peerj.7926)
Supplement: Supplemental Information 1 [file peerj-07-7926-s001.docx]

**Supplementary Information 1. Specimens Examined and Character States.**

**Part 1. List of specimens examined.**

*Anabisetia saldiviai*: PVPH-74, PVPH-75, PVPH-76

*Cerasinops hodgskissi*: MOR 300

*“Laosaurus” minimus*: ROM 46253 (cast)

*Leptoceratops gracilis*: CMN 8889, CMN 8887 (cast)

*Montanoceratops cerorhynchus*: MOR 425

*Prenoceratops pieganensis* - TCM 2003.1.6, TCM 2003.1.2, TCM 2003.1.11, TCM 2003.1.5, TCM 2003.1.1, TCM 2003.1.4, TCM 2003.1.12, TCM 2003.1.3, TCM 2003.1.9, TCM 2003.1.7, TCM 2003.1.8

*Psittacosaurus* sp.: CMN unnumbered cast of IVPP 120888-2

Neornithischia indet: ROM 45971 (cast), ROM 45966 (cast), ROM 45968 (cast), ROM 45961 (cast)

*Orodromeus makelai*: MOR 623B

*Othnielia rex*: ROM 46240 (cast of BYU-ESM-163R)

*Parksosaurus warreni*: ROM 804

*Serendipaceratops arthurcclarkei*: CMN 41628 (cast of NMV O186385)

*Stegoceras validum*: UALVP 2

*Thescelosaurus neglectus*: MOR 979, NCSM 15728

**Part 2. Character statements for the phylogenetic analysis using He et al. (2015) character matrix.**

*Characters 1-162 are identical to those presented by He et al. (2015). New characters are appended to the end of the list.*

1. Head size small relative to body (0) or large relative to body (1).

2. Head shape in dorsal view: elongate, ovoid (0), or triangular, wide over jugals (1).

3. Orbit diameter more than 20% of skull length (0) or less (1).

4. Preorbital region more than 40% (0) or less than 40% (1) the length of the skull.

5. Tip of rostral low and level with maxillary tooth row (1) or raised and dorsal to maxillary tooth row (1).

6. Rostral bone forming beak absent (0) or present (1).

7. Rostral ventral (buccal) process absent (0) or present (1).

8. Anterior face of rostral round, convex (0) or sharply keeled (1).

9. Premaxillary palatal region flat in ventral view (0) or vaulted dorsally (1).

10. Relative height of premaxilla (snout) to orbital region low (0) or deep (1).

11. Premaxilla–prefrontal contact absent (0) or present (1).

12. Convex buccal process anterior to maxillary tooth row formed by premaxilla or premaxilla and maxilla absent (0) or present (1).

13. Premaxilla–maxilla buccal margin relatively straight in ventral view, tooth rows/buccal margins converge rostrally (0) or buccal margin sinuous in ventral view, with premaxillary palatal region flaring widely rostral to tooth row (1).

14. Anterior end of the nasal (internarial bar) above (0) or below and far rostral to the external naris (1).

15. Nares position close to buccal margin (0) or dorsal, away from buccal margin (1) or very far dorsal, level with upper part of orbit (2).

16. Ventral border of external nares significantly below (0), about the level of (1), or significantly above (2) lower rim of infratemporal fenestra.

17. Large depression excavating premaxilla anteroventral to naris absent (0) or present (1).

18. Nasal horn absent (0), small (1), or large (2).

19. Naris width (excluding narial depression) less than 10% of skull length (0) or more than 10% of skull length (1).

20. Position of choana on palate: anterior to maxillary tooth row (0) or level with maxillary tooth row (1).

21. Maxillae from opposite sides separated by vomers at anterior border of the internal choanae (0) or maxillae contact each other anterior to choanae in palatal view and tip of vomer obscured from view (1).

22. Dentigerous margin of maxilla straight (0) or ventrally convex (1).

23. Antorbital fossa reduced or absent (0) or large and triangular or rounded in shape (1).

24. Eminence or tubercle on the rim of the buccal emargination of the maxilla near the junction with the jugal absent (0) or present (1).

25. Palpebral free, articulating with lacrimal (0) or fused to orbital margin (1).

26. Jugal–lacrimal contact reduced (0) or expanded (1).

27. Jugal horns absent (0) or present and laterally directed (1) or present and ventrally directed (2).

28. Jugal suborbital ramus not as deep as subtemporal ramus (0) or suborbital ramus as deep or deeper than orbital ramus (1).

29. Epijugal ossification absent (0) or present (1).

30. Epijugal position on jugal: along dorsal edge of horn (epijugal trapezoidal) (0) or capping end of horn (epijugal conical) (1).

31. Orbital horns absent (0) or present (1).

32. Postorbital inverted L-shaped (0) or triangular and platelike (1).

33. Postorbital with dorsal part rounded and overhanging lateral edge of supratemporal fenestra (0) or with concave dorsal shelf bordering supratemporal fenestra (1).

34. Laterotemporal fenestra with postorbital participation in margin (0), postorbital excluded from margin (1), or jugal–squamosal contact very wide and postorbital situated far from fenestra (2).

35. Laterotemporal fenestra width more than 10% of skull length (0) or less than 10% of skull length (1).

36. Squamosal subtriangular in lateral view (0) or T-shaped, with postquadratic process (1).

37. Temporal process of squamosal simple (0) or deeply bifurcate around temporal process of postorbital (1).

38. Posterior edge of squamosal angled anteromedially (0) or posteromedially, squamosal contributing lateral portion of frill margin (1).

39. Temporal bars of squamosals parallel (0) or posteriorly divergent (1).

40. Quadratojugal mediolaterally flattened (0) or transversely expanded and triangular in coronal section (1) or triangular in coronal section, but with slender anterior prong articulating with jugal (2).

41. Quadrate shaft anteriorly convex in lateral view (0) or straight (1).

42. Elongate parasagittal process of the palatine absent (0) or present (1).

43. Ectopterygoid exposed in palatal view (0) or reduced and concealed in palatal view (1).

44. Ectopterygoid contacts jugal (0) or ectopterygoid reduced and restricted to contact with maxilla (1).

45. Pterygopalatine foramen (modified suborbital fenestra) large (0) or diminutive (1).

46. Ventral ridge on mandibular process of pterygoid defining "Eustachian canal" absent (0) or present (1).

47. Pterygoid–maxilla contact at posterior end of tooth row absent (0) or present (1).

48. Prominent posterior midline process on pterygoid absent (0) or present (1).

49. Pterygoid mandibular process short (0) or long, extending well below maxillary tooth row (1).

50. Pterygoid mandibular process formed only by pterygoid (0) or jointly by pterygoid and ectopterygoid (1).

51. Parieto-frontal contact flat (0), depressed (1), or invaginated by fontanelle (2).

52. Parieto-squamosal frill absent (0) or parietal frill less than 70% of basal length of skull (1) or more than 70% of basal length (2).

53. Dorsal edge of squamosal temporal bar curves medially at the posterior end, arcing confluently into posterior frill margin (0) or dorsal edge of squamosal meets posterior margin of frill at acute angle (1).

54. Frill solid (0) or fenestrated near posterior margin (1).

55. Distinctive indentation on midline of the posterior parietals present (0) or absent (1).

56. Epoccipital ossifications/frill scallops absent (0) or present (1).

57. Basioccipital participates in foramen magnum (0) or basioccipital is excluded from foramen magnum and exoccipitals form less than onethird of condyle (1) or exoccipitals form about half or more of occipital condyle (2).

58. Basioccipital excluded from basal tubera by basisphenoid and limited to occipital midline (0) or basioccipital tubera present (1).

59. Basipterygoid process orientation anterolateral (0), ventral (1), or posteroventral (2) when braincase is oriented with condyle pointing posteriorly.

60. Basioccipital tubera flat, in plane with basioccipital plate (0) or everted posterolaterally, forming lip beneath occipital condyle (1).

61. Notch between posteroventral edge of basisphenoid and base of basipterygoid process deep (0) or notch shallow and base of basipterygoid process close to basioccipital tubera (1).

62. Exoccipital with three exits for cranial nerves X–XII near occipital condyle (0) or with two exits (1).

63. Exoccipital–quadrate separated by ventral flange of squamosal (0) or in contact (1).

64. Paroccipital processes deep (height $ K length) (0) or significantly narrower (1).

65. Supraoccipital participates in dorsal margin of foramen magnum (0) or excluded from foramen magnum by exoccipitals (1).

66. Supraoccipital anteriorly inclined relative to basioccipital (0) or in same plane as posterior face of basioccipital (1).

67. Supraoccipital shape tall, triangular (0) or wider than tall, trapezoid (1) or square (2).

68. Tip of predentary shallow (0) or scooplike (1).

69. Predentary with rounded anterior margin and distally broad posteroventral process (0) or with pointed anterior margin and distally narrow posteroventral process (1).

70. Predentary less than two-thirds of dentary length (0) or equal to or more than two-thirds of dentary length (1).

71. Predentary buccal margin sharp (0) or with a rounded, beveled edge (1) or with grooved, triturating edge (2).

72. Tip of dentary smooth (0) or grooved dorsally for reception of the lateral process of the predentary (1) or bears large pit for reception of the lateral process of the predentary (2).

73. Dentary symphyseal area small (0) or large, forming strong immobile bond with participation of splenial (1).

74. Diastema between predentary and first dentary tooth absent (0) or present (1).

75. Ventral margin of dentary curved (0) or straight (1) in lateral view.

76. Dentary flange absent (0) or present along ventral edge (1).

77. Prominent medial expansion of the central mandible in the middle of the tooth row formed by wide Meckelian groove separating tooth-bearing part of the jaw from external surface absent (0) or present (1).

78. Labial face of dentary smooth below tooth row (0) or rugose and sculpted (1).

79. Contact between dentary and prearticular absent (0) or present (1).

80. Posterior end of splenial simple or with shallow dent (0) or with bifid overlap of angular (1).

81. Distal end of coronoid process rounded (0) or with anterior expansion (1).

82. Coronoid process positioned close to main axis of dentary and posterior to tooth row (0) or set0 lateral to tooth row, and end of tooth row covered by anterior part of coronoid process (1) or tooth row level with posterior edge of coronoid process (2).

83. Coronoid straplike and with subequal depth throughout (0) or with lobate, highly expanded dorsal end much deeper than ventral end that slots between splenial and dentary (1).

84. Surangular without distinct lateral ridge or shelf overhanging angular (0) or shelf/ridge present (1).

85. Lateral surface of surangular flat or only weakly convex (0) or with pronounced laterally convex curvature (in the transverse plane) between the coronoid process and glenoid region (1).

86. Tab on surangular forming lateral wall to glenoid cotyle absent (0) or present (1).

87. Angular without one or more small, lateral tubercles along ventral rim below glenoid articulation (0) or tubercles present (1).

88. Lateral surface of angular flat or slightly convex (0) or angular bears a raised emargination along posteroventral margin of mandible, lateral surface distinctly concave (1).

89. Angular-surangular-dentary contact triradiate (0) or surangular with long ventral process overlapping angular, dentary-surangular and angular-surangular sutures form acute angle on lateral face of mandible (1).

90. Mandibular glenoid narrow and flush with medial margin of surangular flange in dorsal view (0) or glenoid region medially expanded and forming lingual process in dorsal view (1).

91. Surface of prearticular and articular below glenoid smooth (0) or with wide, semicircular ventral process near medial face of glenoid (1).

92. Retroarticular process long (0) or short or absent (1).

93. Three or more teeth in premaxilla (0) or two teeth in premaxilla (1) or 1 tooth in premaxilla (2) or premaxilla edentulous (3).

94. Premaxillary teeth with carinae, and in some cases serrations (0) or premaxillary teeth peglike, crown without carinae (1).

95. Teeth with single roots (0) or with double roots (1).

96. Cheek teeth spaced (0) or closely apressed with determinate eruption and replacement pattern (1).

97. Teeth occlude at an oblique angle (0) or at a vertical angle (1) or at a vertical angle, but dentary teeth have a horizontal shelf on the labial face (2).

98. Teeth without distinct median primary ridge (0) or with very weak and wide median ridge on at least some maxillary teeth (1) or all maxillary and dentary teeth with distinct primary ridge (2).

99. Base of primary ridge confluent with the cingulum on maxillary teeth (0) or base of primary ridge set back from cingulum, which forms a continuous ridge at the crown base (1).

100. Pronounced cingula on cheek teeth absent (0) or present (1).

101. Tooth row double, with only one replacement tooth present at a time (0) or battery-like with multiple (≥3) rows of replacement teeth (1).

102. Both lingual and buccal sides of teeth covered with enamel (0) or enamel restricted to lateral side of maxillary and medial side of dentary teeth (1).

103. Dentary tooth crowns with continuous, smooth root crown transition (0) or bulbous expansion at root-crown transition on labial side of tooth, sometimes worn to form notch or shelf (1).

104. Number of alveoli in dentary less than 20 (0) or more than 20 (1).

105. Cheek teeth with cylindrical roots (0) or roots with mesial and distal faces flattened to slightly grooved (1).

106. Tooth crowns radiate or pennate in lateral view (0) or crowns ovate in lateral view (1).

107. Atlas intercentrum semicircular (0) or disc shaped (1).

108. Atlas intercentrum not fused to odontoid (0) or fused (1).

109. Atlas neurapophyses free (0) or fused to intercentrum/odontoid (1).

110. Axial neural spine low (0) or tall and hatchetshaped (1) or elongate and posteriorly inclined (2).

111. The neural spine of the axis anteroposteriorly short (0) or long, extending caudally to the posterior end of the centrum of the succeeding cervical (1).

112. Syncervical absent (0), partially fused (centra but not arches) (1), or completely coossified (2).

113. Dorsal vertebrae with flat articulations on zygapophyses (0) or tongue and grooves articulations on zygapophyses (1).

114. Number of sacrals: five (0) or six (1) or seven (2) or eight or more (3).

115. Outline of sacrum defines rectangle or hourglass in dorsal view (0) or oval in dorsal view (1).

116. Caudal neural spines short and inclined (0) or tall and straight (1).

117. Tail terminates with series of cylindrical caudals that are devoid of neural spines and chevrons (0) or neural spines and chevrons persist virtually to the end of tail (1).

118. Distal chevrons with lobate expanded shape (0) or rodlike (1).

119. Clavicles absent (0) or present (1).

120. Scapula distinctly curved in sagittal view (0) or relatively flat (1).

121. Scapular blade at acute angle relative to glenoid (0) or almost perpendicular to glenoid (1).

122. Coracoid with smooth, arcuate anterior portion (0) or bearing large anterolateral ridge near confluence of anterior and ventral margins (1).

123. Olecranon process relatively small (0) or enlarged (≥1/3 of ulnar length) (1).

124. More than two distal carpals (0) or less than two distal carpals (1).

125. Manus much smaller than pes (0) or closer to pes in size (1).

126. Shaft of postpubis round (0) or mediolaterally flattened, bladelike (1) in cross section.

127. Postpubis long and ventrally oriented (0) or short and posteriorly directed (1).

128. Prepubis short and rod-shaped (0) or long and flared at anterior end (1).

129. Ischial shaft straight (0) or with posterodorsally convex curvature (1).

130. Femoral fourth trochanter triangular and pendant (0) or parallelogram-shaped and pedant (1) or ridge-like (2) or reduced (3)

131. Tibio-femoral ratio more than one (0) or less than one (1).

132. Foot gracile with long, constricted metatarsus, elongate phalanges (0) or short and uncompressed, all phalanges wider than long (1).

133. Pedal unguals pointed (0) or rounded, hooflike (1).

134. Postorbital lateral surface smooth (0) or strongly sculptured (1).

135. Angular lateral surface smooth (0) or strongly sculptured (1).

136. Rostral end of quadratojugal contacting jugal undivided (0) or bifid around caudal end of jugal (1).

137. Nasals flat or convex on midline (0) or with distinct nasal midline depression (1).

138. Parietal roof flat or gently convex (0) or with sharp midline crest (1).

139. Surangular without a lateral process below glenoid (0) or surangular knob (1).

140. Height of middle caudal neural spine less than or equal to 2 (0), 2.1 to 3 times (1), 3.1 to 4 times (2) or more than 4.1 times (3) the height of the associated centrum.

141. Ridge along the caudoventral edge of squamosal absent (0) or present (1).

142. Occipital surface of supraoccipital flat, convex, or with midline ridge (0) or with midline depression along base of midline ridge (1).

143. Accessory antorbital fenestra between naris and antorbital fenestra absent (0) or present (1).

144. Radius without lateral and medial tuberosities along distal half of shaft (0) or tuberosities present (1).

145. Coronoid process notch along caudal edge of dentary coronoid process absent (0) or present (1).

146. Coronoid process notch wide (0) or constricted notch (1).

147. Cervical centra with ventral keels (0) or some or all postaxial centra without keels (1).

148. Width of proximal end of ungual relative to width of distal end of the preceding phalanx equal (0) or wider (1).

149. Height of the caudal neural spine equal to or less than the length of the corresponding chevron (0) or greater (1).

150. Anteroventral chin absent (0), poorly developed (1) or well-developed (2).

151. Base (lower half) of the coronoid process vertical (0) or steeply inclined (>40%).

152. Circumnarial depression, if deep, simple (0) or complex (1).

153. Narial spine absent (0) or present (1).

154. The dorsal border of iliac blade of iliums erect (0) or strongly lateral eversion (1)

155. The elongation of proximal caudaul neural spines absent (0) or yes (1).

156. Ossified tendons, arrangement: longitudinally arranged (0); basket-like arrangement of fusiform tendons in caudal region (1); double-layered lattice (2).

157. External naris, shape: (0)elliptical; (1)round.

158. Premaxilla, shape in lateral view, except for the processes; 0: longer than high, 1: higher than long

159. Premaxilla, ventral border: 0: flat, 1: convex.

160. Predentary shape: caudal border straight: (0), slightly divided (1), very divided (2).

161. Rostral with a distinct buccal process (0) or expanded and having a straight border between dorsal and buccal processes, covering a large part of the premaxilla (1).(ontogenetically variable, code only on adults)

162. Ventral margin of rostral bone flat or slightly downcurved (0), or tip of rostral stongly pendant (1).

NEW CHARACTERS

163. Distal end of ulna, in medial or lateral view: straight (0), bowed medially (1).

164. Astragalus and tibia: not coossified (0), coossified (1)

165. Penultimate pedal phalanx: length is shorter than preceding pedal phalanx (0), length is subequal to preceding phalanx (1).

**Part 3. Character statements for the phylogenetic analysis using Morschhauser et al. (2019) character matrix.**
